# 2,3-Difluoro-*N*-(2-pyrid­yl)benzamide

**DOI:** 10.1107/S1600536808038269

**Published:** 2008-11-20

**Authors:** John F. Gallagher, Joyce McMahon, Frankie P. Anderson, Alan J. Lough

**Affiliations:** a School of Chemical Sciences, Dublin City University, Dublin 9, Ireland; b School of Chemical Sciences, National Institute for Cellular Biotechnology, Dublin City University, Dublin 9, Ireland; c Department of Chemistry, 80 St George Street, University of Toronto, Toronto, Ontario, Canada M5S 3H6

## Abstract

The title compound, C_12_H_8_F_2_N_2_O, crystallizes with two independent mol­ecules in the asymmetric unit. The independent mol­ecules differ slightly in conformation; the dihedral angles between the benzene and pyridine rings are 51.58 (5) and 49.97 (4)°. In the crystal structure, mol­ecules aggregate *via* N—H⋯N_pyridine_ inter­actions as hydrogen-bonded dimers with the structural motif *R*_2_^2^(8), and these dimers are linked *via* C—H⋯O inter­actions to form a supra­molecular chain.

## Related literature

For background information, see: Chopra & Row (2008 ▶); Donnelly *et al.* (2008 ▶); Gelbrich *et al.* (2007 ▶); McMahon *et al.* (2008 ▶). For a related structure, see: Forbes *et al.* (2001 ▶). For the Cambridge Structural Database, see: Allen (2002 ▶).
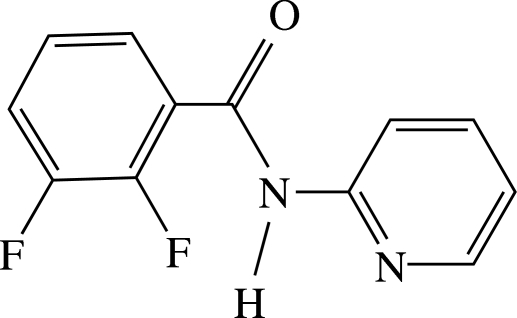


## Experimental

### 

#### Crystal data

C_12_H_8_F_2_N_2_O
*M* *_r_* = 234.20
Monoclinic, 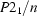

*a* = 11.8515 (4) Å
*b* = 9.0554 (2) Å
*c* = 20.1075 (7) Å
β = 100.2620 (15)°
*V* = 2123.42 (11) Å^3^
*Z* = 8
Mo *K*α radiation
μ = 0.12 mm^−1^
*T* = 150 (1) K
0.26 × 0.20 × 0.15 mm

#### Data collection

Nonius KappaCCD diffractometer
Absorption correction: multi-scan (*SORTAV*; Blessing, 1995 ▶) *T*_min_ = 0.875, *T*_max_ = 0.981
5113 measured reflections
4803 independent reflections
3170 reflections with *I* > 2σ(*I*)
*R*_int_ = 0.045

#### Refinement

*R*[*F* ^2^ > 2σ(*F* ^2^)] = 0.046
*wR*(*F* ^2^) = 0.128
*S* = 1.04
4803 reflections
316 parameters
H atoms treated by a mixture of independent and constrained refinement
Δρ_max_ = 0.22 e Å^−3^
Δρ_min_ = −0.23 e Å^−3^

### 

Data collection: *KappaCCD Server Software* (Nonius, 1997 ▶); cell refinement: *DENZO*–*SMN* (Otwinowski & Minor, 1997 ▶); data reduction: *DENZO*–*SMN*; program(s) used to solve structure: *SHELXS97* (Sheldrick, 2008 ▶); program(s) used to refine structure: *SHELXL97* (Sheldrick, 2008 ▶) and *SORTX* (McArdle, 1995 ▶); mol­ecular graphics: *PLATON* (Spek, 2003 ▶); software used to prepare material for publication: *SHELXL97* and *PREP8* (Ferguson, 1998 ▶).

## Supplementary Material

Crystal structure: contains datablocks global, I. DOI: 10.1107/S1600536808038269/tk2329sup1.cif

Structure factors: contains datablocks I. DOI: 10.1107/S1600536808038269/tk2329Isup2.hkl

Additional supplementary materials:  crystallographic information; 3D view; checkCIF report

## Figures and Tables

**Table 1:** Hydrogen-bond geometry (Å, °)

*D*—H⋯*A*	*D*—H	H⋯*A*	*D*⋯*A*	*D*—H⋯*A*
N1*A*—H1*A*⋯N22*B*	0.894 (19)	2.076 (19)	2.968 (2)	175.9 (16)
N1*B*—H1*B*⋯N22*A*	0.90 (2)	2.10 (2)	2.999 (2)	175.4 (17)
C25*B*—H25*B*⋯O1*A*^i^	0.95	2.48	3.379 (2)	159
C25*A*—H25*A*⋯O1*B*^ii^	0.95	2.67	3.542 (2)	153

Symmetry codes: (i) 
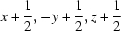
; (ii) 
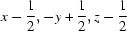
.

**Fig. 1.**
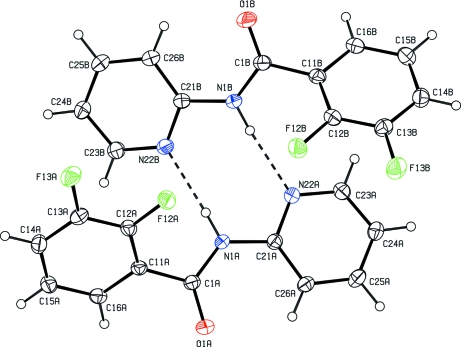
A view of the hydrogen bonded dimeric unit in (I) with the atomic numbering scheme for the two independent molecules A and B. Displacement ellipsoids are drawn at the 30% probability level.

**Fig. 2.**
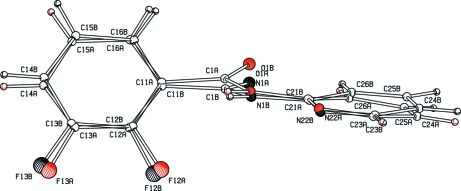
An overlay of the non-H atoms in molecules A and B in (I).

**Fig. 3.**
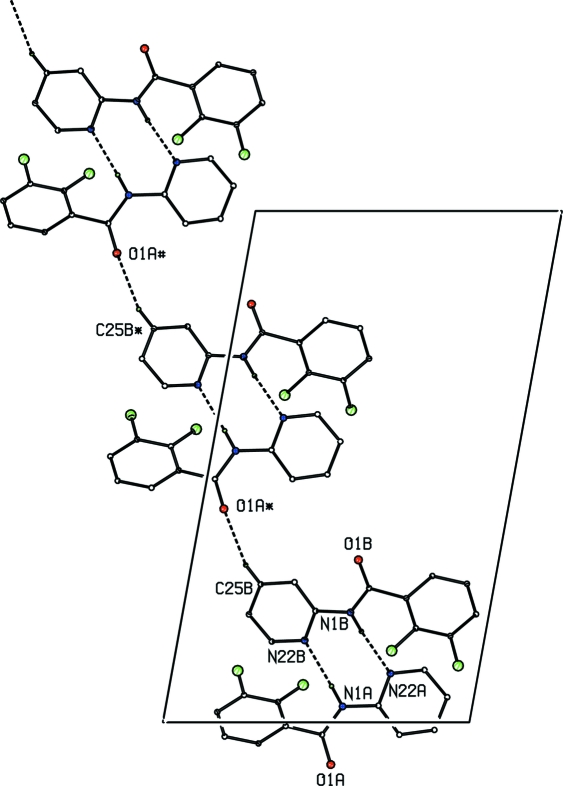
A view of the primary interactions in the crystal structure of (I) with H atoms not involved in hydrogen bonding removed for clarity. Molecules in the hydrogen bonded dimer with suffixes A and B are linked to symmetry related dimers at positions * and # via C—H···O interactions.

## supplementary crystallographic information

### Comment

Our group is completing a structural systematic study of fluoro-*N*'-(pyridyl)benzamide isomers (Donnelly *et al.*, 2008) and are adding to our research with the analogous difluoro-*N*-(pyridyl)benzamide series (McMahon *et al.*, 2008) (Scheme 1). A total of 18 isomers are possible *via* amide formation and resulting through condensation of the 2,3-, 2,4-, 2,5-, 2,6-, 3,4-, 3,5-difluorobenzoyl chlorides with the 4-/3-/2-aminopyridines. The 2,3-, 2,4- and 2,5-difluoro-*N*-(4-pyridyl)benzamides have already been reported by us (McMahon *et al.*, 2008). Systematic structural analyses have recently been reported for related fluoro derivatives (Chopra & Row, 2008) and isomeric series (Gelbrich *et al.*, 2007).

There is a dearth of structural information in the literature on all six possible difluorobenzene derivatives F_2_C_6_H_3_—*Z* (*Z* = remainder of molecule) from analysis of structural data in the Cambridge Structural Database (Allen 2002; v5.29, Nov 2007 issue + 2 updates). In this structural report the structure of 2,3-difluoro-*N*-(2-pyridyl)benzamide (I), Fig. 1, is described.

Compound (I) crystallizes with two molecules, A and B (which differ slightly in conformation) in the asymmetric unit: the C_6_/C_5_N internal angles are 51.58 (5)° and 49.97 (4)°, respectively, see overlay diagram, Fig. 2. Molecules aggregate *via* N—H···N interactions as hydrogen bonded dimers with structural motif *R*_2_^2^(8); see Table 1 for geometric parameters. The [N1A/C21A/N22A/H1A] and [N1B/C21B/N22B/H1B] interplanar angle is 36.2 (3)° and deviates considerably from co-planarity therefore highlighting a degree of twist between the two interacting molecules. Hydrogen bonded dimers are linked into a supramolecular chain *via* C—H···O=C intermolecular interactions, Table 1 and Fig. 3.

An analysis of the Cambridge Structural Database reveals a related structure pentafluoro-*N*-(2-pyridyl)benzamide (II) [CSD code IDALAA] (Forbes *et al.*, 2001) where molecules also form hydrogen bonded dimers in space group *P*1 (No. 2) with *Z*'=2. The N···N intermolecular distances in (II) are 2.9568 (14) and 3.0734 (15) Å.

### Experimental

Compound (I) was synthesized *via* standard condensation procedures and similar to the related syntheses reported previously (Donnelly *et al.*, 2008; McMahon *et al.*, 2008).

Typical organic workup and washing gave the product (I) in modest yield of 15–20%. Crystals suitable for diffraction were grown from CHCl_3_ solution as colourless blocks over a period of 1–2 weeks. The compounds gave clean ^1^H and ^13^C NMR spectra in δ_6_-DMSO and infrared spectra (in CHCl_3_ solution, and as KBr disks).

For (I), m.p. 348–352 K (uncorrected). IR (ν_C=O_ cm^-1^): 1644(*s*), (CHCl_3_); 1695(*s*) (KBr). ^1^H NMR (400 MHz, DMSO): δ 11.02 (s, 1H, N—H), 8.38 (d, 1H), 8.18 (d, 1H), 7.87 (t, 1H), 7.61 (q, 1H), 7.50 (t, 1H), 7.34 (q, 1H), 7.19 (t, 1H).

### Refinement

H atoms attached to C atoms were treated as riding with C—H = 0.95 Å, and with *U*_iso_(H) = 1.2*U*_eq_(C). N-bound H atoms were refined freely with isotropic displacement parameters to bond lengths of 0.894 (19) (for N1—H1A) and 0.90 (2) Å (for N2—H2A).

### Crystal data

**Table:**

C_12_H_8_F_2_N_2_O	*F*_000_ = 960
*M**_r_* = 234.20	*D*_x_ = 1.465 Mg m^−^^3^
Monoclinic, *P*2_1_/*n*	Melting point: 350 K
Hall symbol: -P 2yn	Mo *K*α radiation λ = 0.71073 Å
*a* = 11.8515 (4) Å	Cell parameters from 19375 reflections
*b* = 9.0554 (2) Å	θ = 2.6–27.5º
*c* = 20.1075 (7) Å	µ = 0.12 mm^−^^1^
β = 100.2620 (15)º	*T* = 150 (1) K
*V* = 2123.42 (11) Å^3^	Block, colorless
*Z* = 8	0.26 × 0.20 × 0.15 mm

### Data collection

**Table:**

Nonius KappaCCD diffractometer	4803 independent reflections
Radiation source: fine-focus sealed X-ray tube	3170 reflections with *I* > 2σ(*I*)
Monochromator: graphite	*R*_int_ = 0.045
*T* = 150(1) K	θ_max_ = 27.4º
φ, ω scans with κ offsets	θ_min_ = 2.9º
Absorption correction: multi-scan(SORTAV; Blessing, 1995)	*h* = −15→15
*T*_min_ = 0.875, *T*_max_ = 0.981	*k* = −11→11
5113 measured reflections	*l* = −25→26

### Refinement

**Table:**

Refinement on *F*^2^	Hydrogen site location: inferred from neighbouring sites
Least-squares matrix: full	H atoms treated by a mixture of independent and constrained refinement
*R*[*F*^2^ > 2σ(*F*^2^)] = 0.046	*w* = 1/[σ^2^(*F*_o_^2^) + (0.0702*P*)^2^ + 0.0466*P*] where *P* = (*F*_o_^2^ + 2*F*_c_^2^)/3
*wR*(*F*^2^) = 0.128	(Δ/σ)_max_ < 0.001
*S* = 1.04	Δρ_max_ = 0.22 e Å^−^^3^
4803 reflections	Δρ_min_ = −0.23 e Å^−^^3^
316 parameters	Extinction correction: SHELXL97 (Sheldrick, 2008), Fc^*^=kFc[1+0.001xFc^2^λ^3^/sin(2θ)]^-1/4^
Primary atom site location: structure-invariant direct methods	Extinction coefficient: 0.0082 (18)
Secondary atom site location: difference Fourier map	

### Fractional atomic coordinates and isotropic or equivalent isotropic displacement parameters (Å^2^)

**Table:**

	*x*	*y*	*z*	*U*_iso_*/*U*_eq_	
F12A	0.56446 (8)	0.44877 (11)	0.07324 (5)	0.0425 (3)	
F13A	0.78621 (9)	0.51655 (11)	0.10099 (5)	0.0488 (3)	
O1A	0.42720 (10)	0.20016 (14)	−0.08253 (6)	0.0395 (3)	
C1A	0.47361 (14)	0.22193 (17)	−0.02420 (8)	0.0305 (4)	
N1A	0.42289 (12)	0.20469 (17)	0.03102 (7)	0.0335 (3)	
C11A	0.59705 (14)	0.26753 (17)	−0.00730 (8)	0.0290 (4)	
C12A	0.63682 (14)	0.37604 (18)	0.03976 (8)	0.0321 (4)	
C13A	0.75099 (15)	0.41319 (18)	0.05327 (9)	0.0355 (4)	
C14A	0.82865 (15)	0.3464 (2)	0.02003 (9)	0.0393 (4)	
C15A	0.79011 (15)	0.2389 (2)	−0.02769 (9)	0.0381 (4)	
C16A	0.67583 (15)	0.20015 (18)	−0.04120 (8)	0.0325 (4)	
C21A	0.30662 (14)	0.18055 (17)	0.03269 (8)	0.0299 (4)	
N22A	0.28635 (11)	0.16084 (15)	0.09545 (7)	0.0314 (3)	
C23A	0.17744 (14)	0.13679 (18)	0.10248 (9)	0.0346 (4)	
C24A	0.08688 (15)	0.13592 (19)	0.04919 (9)	0.0377 (4)	
C25A	0.10971 (15)	0.1603 (2)	−0.01501 (10)	0.0410 (4)	
C26A	0.22090 (15)	0.18089 (18)	−0.02435 (9)	0.0350 (4)	
F12B	0.29582 (9)	0.49048 (12)	0.14007 (5)	0.0443 (3)	
F13B	0.06988 (10)	0.53646 (13)	0.11073 (6)	0.0554 (3)	
O1B	0.45792 (11)	0.38227 (17)	0.31766 (6)	0.0555 (4)	
C1B	0.40611 (15)	0.34390 (19)	0.26233 (8)	0.0354 (4)	
N1B	0.45292 (12)	0.27166 (16)	0.21504 (7)	0.0325 (3)	
C11B	0.27918 (14)	0.37060 (18)	0.24278 (8)	0.0328 (4)	
C12B	0.22995 (15)	0.44225 (18)	0.18387 (8)	0.0345 (4)	
C13B	0.11332 (16)	0.46598 (19)	0.16891 (9)	0.0386 (4)	
C14B	0.04218 (16)	0.4186 (2)	0.21145 (10)	0.0421 (4)	
C15B	0.08962 (16)	0.3480 (2)	0.27087 (10)	0.0445 (5)	
C16B	0.20703 (16)	0.3250 (2)	0.28624 (9)	0.0395 (4)	
C21B	0.56780 (14)	0.22849 (17)	0.21788 (8)	0.0303 (4)	
N22B	0.58589 (12)	0.16736 (16)	0.16024 (7)	0.0344 (3)	
C23B	0.69244 (15)	0.1202 (2)	0.15795 (9)	0.0384 (4)	
C24B	0.78295 (15)	0.1326 (2)	0.21110 (9)	0.0389 (4)	
C25B	0.76225 (16)	0.1964 (2)	0.27011 (9)	0.0427 (5)	
C26B	0.65348 (15)	0.2447 (2)	0.27418 (9)	0.0390 (4)	
H1A	0.4709 (16)	0.1978 (19)	0.0706 (9)	0.035 (5)*	
H14A	0.9074	0.3732	0.0295	0.047*	
H15A	0.8428	0.1916	−0.0512	0.046*	
H16A	0.6505	0.1264	−0.0741	0.039*	
H23A	0.1621	0.1195	0.1466	0.042*	
H24A	0.0108	0.1191	0.0563	0.045*	
H25A	0.0486	0.1628	−0.0527	0.049*	
H26A	0.2384	0.1949	−0.0682	0.042*	
H1B	0.4059 (17)	0.240 (2)	0.1778 (10)	0.043 (5)*	
H14B	−0.0383	0.4340	0.2003	0.051*	
H15B	0.0418	0.3153	0.3011	0.053*	
H16B	0.2388	0.2770	0.3273	0.047*	
H23B	0.7063	0.0758	0.1174	0.046*	
H24B	0.8573	0.0983	0.2073	0.047*	
H25B	0.8227	0.2069	0.3077	0.051*	
H26B	0.6375	0.2881	0.3145	0.047*	

### Atomic displacement parameters (Å^2^)

**Table:**

	*U*^11^	*U*^22^	*U*^33^	*U*^12^	*U*^13^	*U*^23^
F12A	0.0384 (6)	0.0438 (6)	0.0455 (6)	0.0052 (4)	0.0084 (5)	−0.0136 (5)
F13A	0.0455 (7)	0.0452 (6)	0.0519 (7)	−0.0065 (5)	−0.0018 (5)	−0.0134 (5)
O1A	0.0386 (7)	0.0519 (8)	0.0264 (7)	−0.0028 (5)	0.0015 (5)	−0.0038 (5)
C1A	0.0317 (9)	0.0328 (9)	0.0258 (9)	0.0030 (7)	0.0020 (7)	−0.0014 (7)
N1A	0.0241 (8)	0.0508 (9)	0.0244 (8)	−0.0028 (6)	0.0008 (6)	−0.0018 (6)
C11A	0.0311 (9)	0.0313 (8)	0.0243 (8)	0.0017 (6)	0.0040 (7)	0.0021 (7)
C12A	0.0321 (9)	0.0333 (9)	0.0315 (9)	0.0058 (7)	0.0073 (7)	−0.0006 (7)
C13A	0.0371 (10)	0.0326 (9)	0.0342 (9)	−0.0036 (7)	−0.0008 (8)	−0.0017 (8)
C14A	0.0286 (9)	0.0461 (10)	0.0428 (11)	−0.0013 (8)	0.0055 (8)	0.0042 (9)
C15A	0.0334 (10)	0.0468 (10)	0.0352 (10)	0.0067 (8)	0.0093 (8)	0.0025 (8)
C16A	0.0373 (10)	0.0337 (9)	0.0266 (9)	0.0030 (7)	0.0056 (7)	0.0006 (7)
C21A	0.0286 (9)	0.0308 (8)	0.0296 (9)	0.0011 (6)	0.0035 (7)	−0.0006 (7)
N22A	0.0286 (8)	0.0353 (8)	0.0300 (8)	−0.0001 (6)	0.0047 (6)	−0.0010 (6)
C23A	0.0357 (10)	0.0339 (9)	0.0354 (10)	−0.0019 (7)	0.0095 (8)	−0.0022 (7)
C24A	0.0296 (9)	0.0401 (10)	0.0426 (11)	−0.0049 (7)	0.0041 (8)	−0.0015 (8)
C25A	0.0322 (10)	0.0476 (11)	0.0389 (10)	−0.0033 (8)	−0.0048 (8)	−0.0009 (8)
C26A	0.0320 (10)	0.0417 (10)	0.0292 (9)	−0.0011 (7)	−0.0003 (7)	0.0016 (8)
F12B	0.0424 (6)	0.0497 (6)	0.0426 (6)	0.0017 (5)	0.0121 (5)	0.0126 (5)
F13B	0.0462 (7)	0.0643 (7)	0.0531 (7)	0.0097 (5)	0.0014 (5)	0.0206 (6)
O1B	0.0459 (8)	0.0824 (10)	0.0345 (8)	0.0130 (7)	−0.0024 (6)	−0.0221 (7)
C1B	0.0376 (10)	0.0408 (10)	0.0272 (9)	0.0025 (7)	0.0041 (8)	−0.0026 (8)
N1B	0.0296 (8)	0.0422 (8)	0.0243 (7)	0.0018 (6)	0.0008 (6)	−0.0035 (6)
C11B	0.0360 (10)	0.0340 (9)	0.0286 (9)	0.0021 (7)	0.0060 (7)	−0.0057 (7)
C12B	0.0374 (10)	0.0356 (9)	0.0320 (9)	−0.0011 (7)	0.0103 (8)	−0.0003 (8)
C13B	0.0389 (11)	0.0376 (10)	0.0377 (10)	0.0043 (8)	0.0022 (8)	0.0030 (8)
C14B	0.0336 (10)	0.0465 (10)	0.0459 (11)	0.0018 (8)	0.0063 (8)	−0.0054 (9)
C15B	0.0417 (11)	0.0544 (11)	0.0402 (11)	−0.0027 (9)	0.0148 (9)	−0.0056 (9)
C16B	0.0420 (11)	0.0489 (11)	0.0284 (9)	0.0032 (8)	0.0084 (8)	−0.0017 (8)
C21B	0.0330 (9)	0.0310 (8)	0.0266 (9)	0.0001 (6)	0.0043 (7)	0.0011 (7)
N22B	0.0331 (8)	0.0393 (8)	0.0292 (8)	0.0037 (6)	0.0015 (6)	−0.0024 (6)
C23B	0.0364 (10)	0.0424 (10)	0.0355 (10)	0.0076 (8)	0.0045 (8)	−0.0038 (8)
C24B	0.0303 (10)	0.0458 (10)	0.0387 (10)	0.0035 (7)	0.0014 (8)	0.0024 (8)
C25B	0.0350 (10)	0.0552 (12)	0.0342 (10)	−0.0007 (8)	−0.0037 (8)	−0.0006 (9)
C26B	0.0367 (10)	0.0504 (11)	0.0280 (9)	0.0002 (8)	0.0006 (8)	−0.0023 (8)

### Geometric parameters (Å, °)

**Table:**

F12A—C12A	1.3520 (18)	C12B—C13B	1.378 (2)
F13A—C13A	1.3522 (19)	C13B—C14B	1.373 (3)
O1A—C1A	1.2195 (19)	C14B—C15B	1.383 (3)
C1A—N1A	1.363 (2)	C15B—C16B	1.386 (3)
C1A—C11A	1.500 (2)	C21B—N22B	1.336 (2)
N1A—C21A	1.402 (2)	C21B—C26B	1.387 (2)
C11A—C12A	1.387 (2)	N22B—C23B	1.342 (2)
C11A—C16A	1.391 (2)	C23B—C24B	1.377 (2)
C12A—C13A	1.374 (2)	C24B—C25B	1.380 (3)
C13A—C14A	1.371 (2)	C25B—C26B	1.377 (3)
C14A—C15A	1.386 (3)	N1A—H1A	0.894 (19)
C15A—C16A	1.378 (2)	C14A—H14A	0.9500
C21A—N22A	1.338 (2)	C15A—H15A	0.9500
C21A—C26A	1.390 (2)	C16A—H16A	0.9500
N22A—C23A	1.341 (2)	C23A—H23A	0.9500
C23A—C24A	1.375 (2)	C24A—H24A	0.9500
C24A—C25A	1.383 (3)	C25A—H25A	0.9500
C25A—C26A	1.377 (2)	C26A—H26A	0.9500
F12B—C12B	1.3493 (19)	N1B—H1B	0.90 (2)
F13B—C13B	1.351 (2)	C14B—H14B	0.9500
O1B—C1B	1.221 (2)	C15B—H15B	0.9500
C1B—N1B	1.352 (2)	C16B—H16B	0.9500
C1B—C11B	1.505 (2)	C23B—H23B	0.9500
N1B—C21B	1.408 (2)	C24B—H24B	0.9500
C11B—C12B	1.385 (2)	C25B—H25B	0.9500
C11B—C16B	1.390 (2)	C26B—H26B	0.9500
			
O1A—C1A—N1A	125.19 (16)	O1B—C1B—N1B	125.13 (16)
O1A—C1A—C11A	121.14 (15)	O1B—C1B—C11B	120.60 (15)
N1A—C1A—C11A	113.64 (14)	N1B—C1B—C11B	114.22 (14)
C1A—N1A—C21A	128.01 (14)	C1B—N1B—C21B	128.38 (14)
C1A—N1A—H1A	115.5 (11)	C1B—N1B—H1B	118.2 (12)
C21A—N1A—H1A	116.1 (11)	C21B—N1B—H1B	113.3 (12)
C12A—C11A—C16A	117.95 (15)	C12B—C11B—C16B	117.68 (16)
C12A—C11A—C1A	123.29 (15)	C12B—C11B—C1B	123.21 (15)
C16A—C11A—C1A	118.75 (15)	C16B—C11B—C1B	119.09 (15)
F12A—C12A—C11A	121.07 (15)	F12B—C12B—C11B	120.36 (15)
F12A—C12A—C13A	118.17 (15)	F12B—C12B—C13B	118.78 (15)
C13A—C12A—C11A	120.76 (15)	C13B—C12B—C11B	120.86 (16)
F13A—C13A—C12A	118.59 (15)	F13B—C13B—C12B	118.38 (16)
F13A—C13A—C14A	120.15 (16)	F13B—C13B—C14B	120.37 (17)
C14A—C13A—C12A	121.25 (16)	C14B—C13B—C12B	121.25 (17)
C13A—C14A—C15A	118.72 (17)	C13B—C14B—C15B	118.86 (17)
C16A—C15A—C14A	120.42 (16)	C14B—C15B—C16B	119.97 (17)
C15A—C16A—C11A	120.89 (16)	C15B—C16B—C11B	121.37 (17)
N22A—C21A—C26A	123.43 (15)	N22B—C21B—C26B	123.00 (16)
N22A—C21A—N1A	112.70 (14)	N22B—C21B—N1B	112.55 (14)
C26A—C21A—N1A	123.84 (15)	C26B—C21B—N1B	124.44 (15)
C21A—N22A—C23A	117.32 (14)	C21B—N22B—C23B	117.33 (14)
N22A—C23A—C24A	123.44 (16)	N22B—C23B—C24B	123.73 (17)
C23A—C24A—C25A	118.11 (16)	C23B—C24B—C25B	117.90 (17)
C26A—C25A—C24A	120.02 (16)	C26B—C25B—C24B	119.71 (17)
C25A—C26A—C21A	117.62 (16)	C25B—C26B—C21B	118.32 (16)
C13A—C14A—H14A	120.6	C13B—C14B—H14B	120.6
C15A—C14A—H14A	120.6	C15B—C14B—H14B	120.6
C16A—C15A—H15A	119.8	C14B—C15B—H15B	120.0
C14A—C15A—H15A	119.8	C16B—C15B—H15B	120.0
C15A—C16A—H16A	119.6	C15B—C16B—H16B	119.3
C11A—C16A—H16A	119.6	C11B—C16B—H16B	119.3
C23A—C24A—H24A	120.9	N22B—C23B—H23B	118.1
C25A—C24A—H24A	120.9	C24B—C23B—H23B	118.1
C26A—C25A—H25A	120.0	C23B—C24B—H24B	121.0
C24A—C25A—H25A	120.0	C25B—C24B—H24B	121.0
N22A—C23A—H23A	118.3	C26B—C25B—H25B	120.1
C24A—C23A—H23A	118.3	C24B—C25B—H25B	120.1
C25A—C26A—H26A	121.2	C25B—C26B—H26B	120.8
C21A—C26A—H26A	121.2	C21B—C26B—H26B	120.8
			
O1A—C1A—N1A—C21A	−10.0 (3)	O1B—C1B—N1B—C21B	−1.4 (3)
C11A—C1A—N1A—C21A	171.67 (15)	C11B—C1B—N1B—C21B	−179.18 (15)
O1A—C1A—C11A—C12A	137.44 (17)	O1B—C1B—C11B—C12B	127.03 (19)
N1A—C1A—C11A—C12A	−44.2 (2)	N1B—C1B—C11B—C12B	−55.1 (2)
O1A—C1A—C11A—C16A	−41.3 (2)	O1B—C1B—C11B—C16B	−51.4 (2)
N1A—C1A—C11A—C16A	137.08 (16)	N1B—C1B—C11B—C16B	126.52 (17)
C16A—C11A—C12A—F12A	178.24 (14)	C16B—C11B—C12B—F12B	179.31 (15)
C1A—C11A—C12A—F12A	−0.5 (2)	C1B—C11B—C12B—F12B	0.9 (2)
C16A—C11A—C12A—C13A	−1.3 (2)	C16B—C11B—C12B—C13B	−0.5 (2)
C1A—C11A—C12A—C13A	179.93 (16)	C1B—C11B—C12B—C13B	−178.94 (16)
F12A—C12A—C13A—F13A	2.4 (2)	F12B—C12B—C13B—F13B	0.2 (2)
C11A—C12A—C13A—F13A	−178.07 (15)	C11B—C12B—C13B—F13B	180.00 (15)
F12A—C12A—C13A—C14A	−178.45 (15)	F12B—C12B—C13B—C14B	179.65 (16)
C11A—C12A—C13A—C14A	1.1 (3)	C11B—C12B—C13B—C14B	−0.5 (3)
F13A—C13A—C14A—C15A	178.79 (16)	F13B—C13B—C14B—C15B	−179.45 (16)
C12A—C13A—C14A—C15A	−0.4 (3)	C12B—C13B—C14B—C15B	1.1 (3)
C13A—C14A—C15A—C16A	−0.1 (3)	C13B—C14B—C15B—C16B	−0.6 (3)
C14A—C15A—C16A—C11A	−0.1 (3)	C14B—C15B—C16B—C11B	−0.5 (3)
C12A—C11A—C16A—C15A	0.8 (2)	C12B—C11B—C16B—C15B	1.0 (3)
C1A—C11A—C16A—C15A	179.64 (15)	C1B—C11B—C16B—C15B	179.48 (16)
C1A—N1A—C21A—N22A	176.76 (16)	C1B—N1B—C21B—N22B	−175.88 (16)
C1A—N1A—C21A—C26A	−4.9 (3)	C1B—N1B—C21B—C26B	5.0 (3)
C26A—C21A—N22A—C23A	1.7 (2)	C26B—C21B—N22B—C23B	0.1 (2)
N1A—C21A—N22A—C23A	−179.97 (14)	N1B—C21B—N22B—C23B	−179.03 (15)
C21A—N22A—C23A—C24A	−2.0 (2)	C21B—N22B—C23B—C24B	−0.5 (3)
N22A—C23A—C24A—C25A	0.4 (3)	N22B—C23B—C24B—C25B	0.4 (3)
C23A—C24A—C25A—C26A	1.6 (3)	C23B—C24B—C25B—C26B	0.2 (3)
C24A—C25A—C26A—C21A	−1.9 (3)	C24B—C25B—C26B—C21B	−0.6 (3)
N22A—C21A—C26A—C25A	0.2 (3)	N22B—C21B—C26B—C25B	0.5 (3)
N1A—C21A—C26A—C25A	−177.96 (16)	N1B—C21B—C26B—C25B	179.45 (16)

### Hydrogen-bond geometry (Å, °)

**Table:**

*D*—H···*A*	*D*—H	H···*A*	*D*···*A*	*D*—H···*A*
N1A—H1A···N22B	0.894 (19)	2.076 (19)	2.968 (2)	175.9 (16)
N1B—H1B···N22A	0.90 (2)	2.10 (2)	2.999 (2)	175.4 (17)
C26A—H26A···O1A	0.95	2.31	2.898 (2)	120
C26B—H26B···O1B	0.95	2.30	2.900 (2)	120
C25B—H25B···O1A^i^	0.95	2.48	3.379 (2)	159
C25A—H25A···O1B^ii^	0.95	2.67	3.542 (2)	153

Symmetry codes: (i) *x*+1/2, −*y*+1/2, *z*+1/2; (ii) *x*−1/2, −*y*+1/2, *z*−1/2.
